# A Review of Fecal Microbiota Transplantation in Children—Exploring Its Role in the Treatment of Inflammatory Bowel Diseases

**DOI:** 10.3390/medicina60111899

**Published:** 2024-11-20

**Authors:** Yanna Ko, Sara Alaedin, Dewni Fernando, Jerry Zhou, Vincent Ho

**Affiliations:** 1 School of Medicine, Western Sydney University, Campbelltown Campus, Sydney, NSW 2747, Australia; yanna.ko@health.nsw.gov.au (Y.K.); 22055379@student.westernsydney.edu.au (S.A.); 19967413@student.westernsydney.edu.au (D.F.); j.zhou@westernsydney.edu.au (J.Z.); 2 Canterbury Hospital, Sydney, NSW 2194, Australia; 3 Camden and Campbelltown Hospitals, Sydney, NSW 2560, Australia

**Keywords:** fecal microbiota transplantation (FMT), inflammatory bowel disease (IBD), ulcerative colitis (UC), Crohn’s disease (CD), gut microbiome, pediatric

## Abstract

*Background and Objectives*: There is an increasing use of fecal matter transplantation (FMT) worldwide as research into the impact of the gut microbiome in various disease states is growing. FMT is the transfer of stool from a healthy human donor to a patient for the purpose of restoring intestinal dysbiosis. This review will assess the efficacy and safety of FMT in the treatment of pediatric inflammatory bowel diseases (IBDs) and explore the future directions of the use of FMT in children. *Materials and Methods*: A systematic review was performed where a literature search of publications published prior to 15 September 2023 was performed. Efficacy outcomes and safety data as well as microbiome analysis were reviewed from the studies where applicable. *Results*: Nine studies on UC and two studies on CD satisfied eligibility criteria and individually analysed. Most of the studies provided microbiome analyses. *Conclusions*: FMT is a safe treatment for paediatric IBD, and is shown to be effective in inducing clinical response by some studies. However the lack of randomized controlled trials limited the results of our study.

## 1. Introduction

Inflammatory bowel disease (IBD) is a chronic inflammatory condition affecting the gastrointestinal tract and is subcategorized into ulcerative colitis (UC) and Crohn’s disease (CD). There is an increasing use of fecal matter transplantation (FMT) worldwide as research into the impact of the gut microbiome in various disease states is growing, including IBD. FMT is the transfer of stool from a healthy human donor to a patient with the intention of restoring intestinal dysbiosis [1]. As FMT involves harvesting and administrating fecal matter from a healthy donor to an individual with a possible dysregulated gut microbiome, it allows for the reconstruction and reconstitution of the intestinal microbiome, leading to the restoration of the immune responses [1]. FMT has become a common treatment for recurrent *Clostridioides difficile* infection [2] and is becoming more frequent in use for adult conditions, such as UC.

Despite the growing number of studies in adult gastrointestinal conditions, the number of placebo-controlled studies in children is small. Exploring the role of FMT in children with CD or UC is important so that clinicians can understand its potential for the treatment of these debilitating conditions, particularly when the microbiome in childhood and adolescence may be more susceptible to external environmental influences. Additionally, there may be a greater potential for FMT to modulate the interaction between the gut microbiome and the environment in children due to the reduced duration of exposure to IBD environmental risk factors. Therefore, the effect of FMT in pediatric IBD may be even greater than that in the adult IBD population, as the pathogenesis of CD and UC is based on a complex interplay between environmental, genetic,, and microbial factors, leading to gut immune dysregulation [3,4,5]. While there have been differences shown in the gut microbiome between children and adults, the efficacy of FMT in the pediatric population with IBD is unclear [6,7,8]. The objective of this systematic review is to assess the current evidence of FMT in the treatment of pediatric IBD regarding safety and efficacy and explore future directions in pediatric uses of FMT.

## 2. Materials and Methods

A systematic review following the methodology described by the Joanna Briggs Institute and using the guidelines of the Preferred Reporting Items for Systematic and Meta-Analysis Extension for Scoping Reviews (PRISMA-ScR) of 2020 was performed.

### 2.1. Inclusion and Exclusion Criteria

The method of fecal matter transplantation included any mode of the introduction of fecal matter, such as enemas, insertion via a nasogastric tube, and a colonoscopy. There were no date restrictions applied to the articles. Only studies written in English evaluating the use of FMT in children and adolescents (aged from 0 to 21 years) were selected. Studies that included the use of FMT in a pediatric patient were included, with a minimum follow-up duration of 2 weeks. Cohort studies and case–control studies with subjects identified as having UC or CD were included without limitation to disease severity or phenotype. Opinion pieces, published abstracts, letters to the editor, studies without clinical data with a specified number of patients, animal studies, and expert reviews were excluded.

### 2.2. Search Strategy

Two authors (Y.K., D.F.) searched for articles on the 15th of September 2023 using the following databases: (1) MEDLINE (Ovid), (2) PubMed, (3) Cochrane Library, (4) Embase, and (5) Web of Science. Search key terms included four main categories: fecal matter transplantation, treatment, pediatric, adolescent, Crohn’s disease, and ulcerative colitis. The full search strategy can be found in the supplementary Table S1.

### 2.3. Study Selection

Endnote software was used to remove duplicate articles. One author reviewed the articles using the titles and abstracts to identify studies for inclusion. The same author then reviewed the full-text versions. To avoid missing any useful studies that could have been missed during the database search, references of relevant studies were manually searched. Due to the significant heterogeneity in FMT administration methods and follow-up durations, it was concluded that a meta-analysis was not feasible.

### 2.4. Risk of Bias

For case–control and cohort studies, the Newcastle–Ottawa Scale (NOS) was used to evaluate the risk of bias in these studies. Studies were categorized as having a low, medium, or high risk of bias using a star system consisting of nine items [9]. The risk of bias in the case series was assessed using the quality assessment tools developed by the US National Heart Lung and Blood Institute (NHLBI) of the National Institutes of Health (NIH) [10]. The tools allowed for the determination of good quality (total score of 7–9), moderate quality (4–6), and poor quality (1–3). A quality appraisal was performed by two authors: Y.K. and D.F. Any disagreements were reviewed by V.H. (see Table 1).

### 2.5. Study Outcomes

The primary outcome was the assessment of the efficacy of FMT in the treatment of pediatric UC and CD, as assessed by clinical remission rates. The secondary outcome was the safety of FMT in the treatment of pediatric IBD patients, as assessed through the event rate of adverse events (AEs), serious adverse events (SAEs), and deaths.

## 3. Results

**Figure 1:**
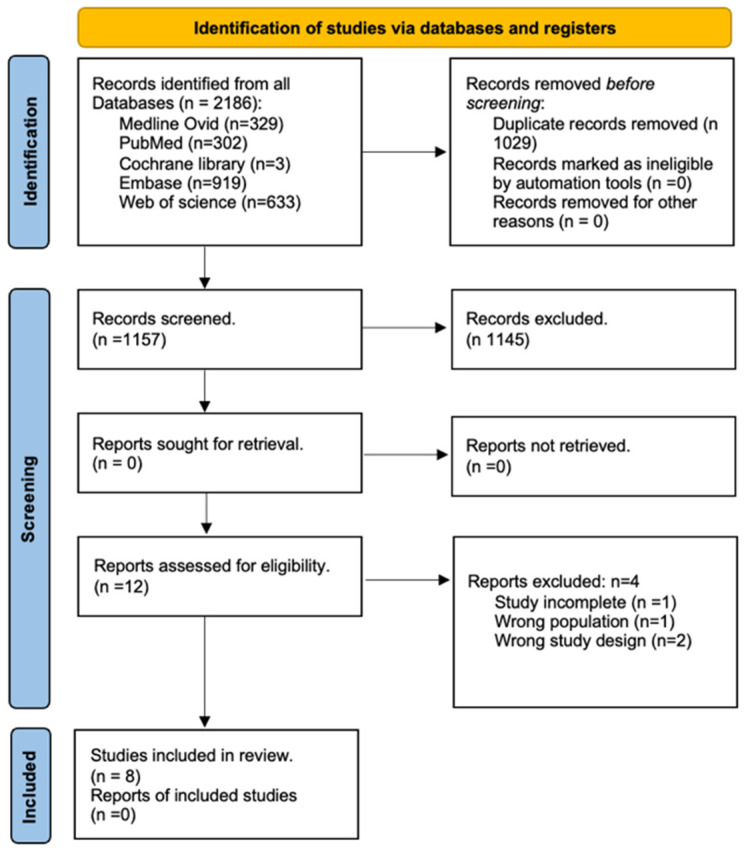
Preferred reporting items for systematic review (PRISMA).

After removing duplicates, the search revealed 2186 studies through databases and registers (Figure 1). Twelve studies were obtained after screening and were reviewed thoroughly to assess their eligibility. Eight studies were finally selected for inclusion in this review.

### 3.1. Fecal Microbiota Transplantation in Pediatric Ulcerative Colitis

UC is an inflammatory condition of the mucosa that tends to commence in the distal region of the colon and can extend to involve the entire colon [6]. UC can exhibit a remitting and relapsing nature [4], and although the condition can affect both adults and the pediatric population, 25% of patients present with childhood-onset ulcerative colitis [3,4,5].

In total, nine studies were included in the systematic review (one uncontrolled trial, two prospective studies, one phase I open-label study, and four case reports) (see Table 2). There was a total of 32 pediatric UC patients studied, with the longest follow-up time of 2 years. The study outcomes evaluated in these studies were efficacy as a clinical response to FMT, endoscopic change, safety and tolerance of FMT, and microbiota modification. The studies included pediatric patients with UC of different severities, lengths of disease, and FMT administration through different routes. Two studies used FMT retention enemas, one used endoscopic instillation, and the rest of the studies utilized mixed methods, including retention enemas, nasoduodenal tubes, and colonoscopic instillations.

The efficacy and clinical response in the studies were determined using the PUCAI score (Pediatric Ulcerative Colitis Activity Index) and some reported responses as assessed through fecal calprotectin levels. The PUCAI score is a measure of the clinical disease activity for each patient. The overall average efficacy of FMT in UC pediatric patients as measured by clinical remission was 28%. The efficacy of FMT may be influenced by baseline disease severity, as the study by Kunde S et al. found FMT in pediatric patients with severe UC had reduced clinical response rates compared to those with mild or moderate severity [14]. Another study also found a lack of clinical response in severe and refractory UC to previous lines of therapy in a pediatric patient administered FMT via the nasoduodenal route [15]. The longest duration of sustained clinical remission to FMT was up to 24 months in the study by Yodoshi et al. [13]. The patient with the prolonged clinical remission also demonstrated early clinical response by week 3 post-FMT. Additionally, there may be increased chances of sustained clinical remission if repeated courses of FMT are administered, as demonstrated by a 100% clinical remission rate in a study that administered FMT in repeated doses over the first ten months of therapy [16].

Two studies compared efficacy in the form of clinical response when comparing the route of administration of FMT [21,22]. A colonoscopy was found to have the highest rates of clinical response in the recipient, followed by retention enema, with nasogastric administration being the least effective [19]. These findings were further supported by rates of clinical remission, which were the highest with endoscopic instillation compared to oral routes of administration [22]. Endoscopic administration may have improved efficacy due to bypassing the lower pH environment of the upper gastrointestinal tract and improved tolerability.

All studies indicated that FMT had reasonable safety, and no immediate or long-term adverse events were reported apart from fever within the first 48 h of FMT administration in two subjects out of thirty-two, with a resolution using antipyretics. In regard to tolerability, one study reported that one child out of nine could not hold the FMT retention enema [14], with the age of the youngest subject being 7 years old No adverse events resulted in hospitalization or death occurred following FMT administration in all of the studies. Overall, adverse effects experienced by the subjects in the studies were found to be self-limiting.

Five studies [12,13,15,16,18] evaluated gut microbiota changes via 16sRNA genome sequencing, of which four studies [12,13,16,18] showed that there were beneficial changes to the microbiome after FMT administration in children, demonstrating increased microbial richness and diversity. One study revealed an increase in microbiota richness due to *Collinsella aerofaciens* and *Eubacterium biforme* [11]. This study also suggested that remission of the patient’s condition may be linked to an increase in species of *Mogibacteriaceae*, *Bacteroides plebeius*, *Parabacteroides*, *Ruminococcus bromii*, *B. Bacteroides ovatus*, and *Bacteroides eggerthi*, along with a decrease in *Blautia producta* and *Proteus species* [11]. Another study comparing pre- and post-FMT microbiomes revealed a significant improvement in alpha diversity of the microbiome post-FMT treatment. The study also revealed very low levels of *Bacterioides* and a high level of *Klebsiella* and *Escherichia* prior to FMT. Additionally, an increase in the numbers of *Coproccus* and *Lachnospiraceae* post-FMT that may have produced beneficial effects was suggested by another study [18]. Furthermore, an open-label study by Nusbaum DJ et al. revealed that the alpha diversity of the gut microbiome increased and shifted towards donor levels following FMT [12]. The metabolomic profile, however, only shifted towards the donor profiles in three of the four subjects who responded to FMT and was less similar in one subject. Quantification of short-chain fatty acids after FMT showed a decrease in acetic acid levels and an increase in butyric acid levels. In a case study of a failed FMT in a three-year-old child, analysis of the microbiome showed that the patient’s microbiome was not like the healthy donor, which may have resulted from the failure to retain the fecal microbiota that was transferred [15]. The authors suggested the failure to retain the transferred microbiota may be due to the severity of the underlying disease, of which the subject had severe UC. After repeated FMT in a child with UC in one case study, an improvement in the diversity of the microbiome was seen with increased *Bacteroides*, *Acidaminococcus*, *Faecaliacterium*, *Bifidobacterium*, and *Eubacterium* by week 3 of the study, which was maintained at week 32 [16]. In studies by Kellermayer R et al. [18] and Quagliariello A et al. [11], participant microbiomes were different from donor microbiomes, but FMT still produced an increase in diversity and richness of the microbiome, as was the case for the other studies, which reported clinical response from FMT.

### 3.2. Fecal Microbiota Transplantation in Pediatric Crohn’s Disease

The number of studies investigating the use of FMT for pediatric CD is smaller compared to those evaluating pediatric UC. Two studies were included that assessed efficacy and safety following FMT in pediatric CD subjects (see Table 3). There was a total of 16 subjects. The CD phenotype was variable, but none of the subjects were recorded to have fistulizing or structuring CD. The method of FMT administration was variable with endoscopic and nasoduodenal routes used. Nine subjects were administered a single FMT via a nasogastric tube. The age ranges of these children were between 8 and 19 years. The studies showed there was evidence of clinical response and remission, as measured by the Pediatric Crohn’s Disease Activity Index score, with an average efficacy of 56.3% for clinical remission in the 16 subjects. Clinical remission was evident for up to 6 months (two subjects). When comparing the two studies, higher clinical remission rates were achieved through the nasoduodenal administration of FMT, which contrasted with the findings of the studies in pediatric UC subjects where endoscopic administration had higher efficacy. This may be related to the different disease characteristics of CD and its transmural inflammation compared to UC.

Both studies found that FMT had reasonable safety with no immediate or long-term adverse events reported, and it was tolerated well.

The two studies also reported an increase in microbial diversity in subjects post-FMT therapy for all clinical responders, as measured by the alpha diversity of the recipient’s gut microbiome. Goyal et al. reported that the alpha diversity of samples at 1 week and 1 month post-FMT administration also approached the level of donor samples, suggesting a rapid response to FMT [20].

## 4. Discussion

Overall, the studies showed that the use of FMT in the treatment of pediatric CD and UC leads to at least moderate efficacy. The studies demonstrated that the use of FMT in pediatric IBD usually resulted in improvements in clinical response and remission, with modifications to the microbiome. The studies also showed that FMT is well tolerated in the pediatric IBD population. The findings of this review support the conclusions of a published meta-analysis in the treatment of IBD using FMT, which reported the achievement of a clinical response in 58.8% of patients within 4 weeks and clinical remission in 64.7% of patients [23]. Additionally, the results presented in this review also support the findings of a published meta-analysis of the treatment UC in adults and children with FMT, in which the persistence of alterations in the gut microbiome with a shift towards donor microbiota was demonstrated [24].

All studies included had small sample sizes and lacked a sham-treated control group. Therefore, the results need to be interpreted with caution, as the true effect of FMT on clinical response and remission cannot be certain. Case reports are considered low-quality evidence according to the hierarchy of research studies; therefore, higher-quality studies are required to assess the effectiveness and safety of FMT in pediatric populations. Moreover, the interventions, characteristics of donors and subjects, and follow-up times were not constant to assess the effectiveness of FMT. These limitations highlight the need for randomized controlled trials of FMT in pediatric IBD patients.

In Suskind et al.’s study, the stool donor for each patient was their biological mother, except for two patients who received donor stools from their biological father [19]. Similarly, Goyal et al.’s study also procured donor stools from the subjects’ family members but also included trusted friends [20]. The selection criteria for donor use highlight an area of uncertainty for FMT use in pediatric IBD. The criteria for donor selection for pediatric use is still uncertain, and the optimal donor is undefined. Thus far, the selection of donors for FMT production has only been studied in adults. Donor–recipient matching models have been investigated in the use of FMT for the treatment of UC in adults, which might enable a more specific transplantation of FMT from a donor suited to the deficiencies in the recipient’s gut microbiota and thus improve response to FMT. This also has the potential to allow greater stability of beneficial species in the recipient microbiome. Additionally, it has been shown that UC patients have lower abundances of *Alistipes*, *Odoribacter*, and *Coprococcus*, which are all associated with short-chain fatty acid production; thus, donors could potentially be selected to ensure the potential to restore these specific microbial communities [25]. These findings will need to be investigated in pediatric populations to better understand their applicability to children.

While effectiveness has been shown in the treatment of pediatric UC, there are areas of uncertainty related to FMT use. One issue is the best form of FMT administration in the setting of fresh versus frozen FMT. In the treatment of recurrent *Clostridioides* difficile, one study showed a lack of statistically significant differences between fresh and frozen FMT in the treatment of the condition [26]; however, no specific pediatric study has addressed differences between fresh and frozen FMT thus far. Frozen FMT is advantageous for the use of pediatric patients, as repeated donor sample collection for fresh samples can be time consuming and potentially require repeated clinic visits. Additionally, other routes, including retention enema, endoscopic instillation, or nasoduodenal tube delivery, are available but have not been studied in a head-to-head trial. Published studies, thus far, have employed major differences in design, which make comparisons difficult. These include the duration of treatment, choice of control group, dose, timing, and mode of FMT. Overall, all methods need to consider the balance between efficacy and tolerability in this special pediatric population.

## 5. Conclusions

FMT holds promise as a therapeutic option for pediatric IBD. Even though studies are limited, they demonstrate positive outcomes in symptom relief and microbiota changes. However, randomized controlled trials are not available to determine the benefits and risks of FMT in a pediatric population. Though this review provides an understanding of the current use and effects of the treatment of FMT in pediatric UC and CD, the data are insufficient to support the recommendation of FMT in pediatric patients for clinical practice. Higher-quality evidence is needed. More clinical trials will be required to assess the safety, efficacy, and tolerability of FMT. Additionally, challenges related to donor selection, placebo effects, and safety considerations necessitate further exploration. Future research should focus on refining treatment protocols, investigating microbial markers, and conducting large-scale trials to establish the efficacy and safety of FMT in the context of IBD in pediatric patients.

## Figures and Tables

**Table 1:** Summary of risk of bias assessment for the included studies.

Author/Year	Study Design	Quality Assessment Tool	Quality Score
Quagliariello A et al. (2020) [11]	Case series	NOS	5
Nusbaum DJ et al. (2018) [12]	Case series	NIH	6
Yodoshi T et al. (2018) [13]	Case series	NIH	5
Kunde S et al. (2013) [14]	Case series	NOS	6
Kumagai H et al. (2016) [15]	Case series	NOS	5
Shimizu H et al. (2016) [16]	Case series	NIH	5
Suskind DL et al. (2015) [17]	Prospective study	NOS	9
Kellermayer R et al. (2015) [18]	Case series	NIH	6
Suskind DL et al. (2015) [19]	Prospective study	NOS	7
Goyal A et al. (2018) [20]	Case series	NOS	9

Newcastle–Ottawa Scale (NOS); National Institutes of Health (NIH).

**Table 2:** Characteristics of studies evaluating fecal microbiota transplantation in pediatric ulcerative colitis.

Study (Year, Reference)	Country	Study Design	Primary Outcome	Intervention FMT and Donor Information If Available	Sample Size	Concomitant Medication	Follow-Up Time Following FMT	Microbial Diversity	Clinical Remission (Ever)
Quagliariello A et al. (2020) [11]	Italy	Case report	Symptom response	Endoscopic instillation Single FMT donor	2	Patient 1—mesalazine Patient 2—mesalazine, azathioprine, and metronidazole	4, 8, 12, 16 weeks and 12 months	Increased in both	1
Nusbaum DJ et al. (2018) [12]	United States	Single-center pilot study—open-label uncontrolled trial	16sRNA microbiome profiling	Retention enema for one hour for five days	9	Nil	4 weeks	Increased in all	Was not assessed
Yodoshi T et al. (2018) [13]	Japan	Case report	Symptom response	Intra-colonoscopic administration or enteral feeding tube via esophagus once per day for 5 days	2	Case 1—azathioprine, cyclosporine, and infliximab Case 2—azathioprine, tacrolimus, and infliximab	3, 4, 8 weeks, 3 months, and 24 months	Increased in one patient	1
Kunde S et al. (2013) [14]	United States	Prospective, open-label, uncontrolled, single-center pilot study	Safety and tolerability	Retention enema for 1 h for 5 days	10	Nil	4 weeks	Not assessed	3
Kumagai H et al. (2016) [15]	Japan	Case report	Symptom response	Six times by retention enema (×2) and via a nasoduodenal tube (×4) within 10 days	1	Infliximab, tacrolimus, probiotic therapy with *Clostridioides butyricum*	2 days	Unchanged	0
Shimizu H et al. (2016) [16]	Japan	Case report	Symptom response	Bowel preparation with magnesium citrate the day before the first FMT delivered via colonoscopy. FMT by retention enema for the next 4 days administered. 11 additional FMT by retention enema every 2–4 weeks over 10 months	1	Infliximab, tacrolimus, and prednisone	40 weeks	Increased	1
Suskind DL et al. (2015) [17]	United States	Prospective open-label study	Symptom response	Nasogastric tube	4	3 subjects were on oral mesalamine, 2 subjects were on VSL#3, and 1 subject was receiving azathioprine	2, 6, 12 weeks	Not assessed	0
Kellermayer R et al. (2015) [18]	United States	Phase 1 open-label study	Symptom response	Colonoscopy and enemas during a 6–12-week period	3	Infliximab, 6-mercaptopurine, and steroids	2, 4 weeks, 105 days	Increased in all	3

**Table 3:** Characteristics of studies evaluating fecal microbiota transplantation in pediatric Crohn’s disease.

Study (Year, Reference)	Country	Study Design	Intervention FMT	Sample Size	Concomitant Medication	Follow-Up Time Following FMT	Microbial Diversity	Clinical Remission (Ever)
Suskind DL et al. (2015) [19]	United States	Prospective, open-label, uncontrolled, single-center pilot study	Single FMT via nasogastric tube	9	Nil	12 weeks	Not assessed	7
Goyal A et al. (2018) [20]	United States	Open-label, single-center prospective trial	Single FMT by upper and lower endoscopy	7	3 subjects were on corticosteroids, 2 subjects were on immunomodulators, 4 subjects were on mesalamine, and 4 subjects were on biologics	6 months	Increased	2

## Data Availability

No new data were created or analyzed in this study. Data sharing is not applicable to this article.

## Supplementary Materials

The following supporting information can be downloaded at: https://www.mdpi.com/article/10.3390/medicina60111899/s1, Table S1. Search Strategy.
