# Supplementary material for: A Review of Fecal Microbiota Transplantation in Children—Exploring Its Role in the Treatment of Inflammatory Bowel Diseases
Source: Medicina (Kaunas). 2024 Nov 20;60(11):1899. doi: 10.3390/medicina60111899 (PMC11596230; doi:10.3390/medicina60111899)
Supplement: Supplementary file 1 [file medicina-60-01899-s001.zip › medicina-3246935-supplementary.pdf]

Supplementary Table S1. Search Strategy.

		Results
MEDLINE (Ovid)	#1 Paediatric<br><br>(Children or "school-aged children" or adolescents or paediatric or pediatric or infants).ti,ab.<br><br>#2 Faecal microbial transplantation<br><br>("Faecal" or FMT or "Fecal" or "microbiota" or "stool" or transplant or transplantation or implant or implantation or feces or faeces or microflora or fecal flora or faecal flora or transfusion or implant or implantation or instillation or donor or bacteriotherapy or infusion).ti,ab.<br><br>#3 treatment<br><br>(treatment or therapeutic or therapy or "reduction in recurrence" or successful or "reduction in rate").ti,ab.<br><br>#4 Ulcerative Colitis<br><br>("ulcerative colitis" or UC).ti,ab.<br><br>#1 and #2 and #3 and #4	329
PubMed	(((Children[Title/Abstract] OR school-aged children[Title/Abstract] OR adolescents[Title/Abstract] OR paediatric[Title/Abstract] OR pediatric[Title/Abstract] OR infants[Title/Abstract]) AND (Faecal[Title/Abstract] OR FMT[Title/Abstract] OR Fecal[Title/Abstract] OR microbiota[Title/Abstract] OR stool[Title/Abstract] OR transplant[Title/Abstract] OR transplantation[Title/Abstract] OR implant[Title/Abstract] OR implantation[Title/Abstract] OR feces[Title/Abstract] OR faeces[Title/Abstract] OR microflora[Title/Abstract] OR fecal flora[Title/Abstract] OR faecal flora[Title/Abstract] OR transfusion[Title/Abstract] OR implant[Title/Abstract] OR implantation[Title/Abstract] OR instillation[Title/Abstract] OR donor[Title/Abstract] OR bacteriotherapy[Title/Abstract] OR infusion[Title/Abstract])) AND (treatment[Title/Abstract] OR therapeutic[Title/Abstract] OR "reduction in recurrence"[Title/Abstract] OR successful[Title/Abstract] OR "reduction in rate"[Title/Abstract])) AND ("ulcerative colitis"[Title/Abstract] OR UC[Title/Abstract])	302

Cochrane Library	Children or "school-aged children" or adolescents or paediatric or pediatric or infants in Title Abstract Keyword AND "Faecal" or FMT or "Fecal" or "microbiota" or "stool" or transplant or transplantation or implant or implantation or feces or faeces or microflora or fecal flora or faecal flora or transfusion or implant or implantation or instillation or donor or bacteriotherapy or infusion in Title Abstract Keyword AND treatment or therapeutic or "reduction in recurrence" or successful or "reduction in rate" in Title Abstract Keyword AND "ulcerative colitis" or UC in Title Abstract Keyword	3
Embase	#1 Paediatric<br><br>(Children or "school-aged children" or adolescents or paediatric or pediatric or infants).ti,ab.<br><br>#2 Faecal microbial transplantation<br><br>("Faecal" or FMT or "Fecal" or "microbiota" or "stool" or transplant or transplantation or implant or implantation or feces or faeces or microflora or fecal flora or faecal flora or transfusion or implant or implantation or instillation or donor or bacteriotherapy or infusion).ti,ab.<br><br>#3 treatment<br><br>(treatment or therapeutic or therapy or "reduction in recurrence" or successful or "reduction in rate").ti,ab.<br><br>#4 Ulcerative Colitis<br><br>("ulcerative colitis" or UC).ti,ab.<br><br>#1 and #2 and #3 and #4	919
Web of Science	(((TS=(Children or "school-aged children" or adolescents or paediatric or pediatric or infants)) AND TS=("Faecal" or FMT or "Fecal" or "microbiota" or "stool" or transplant or transplantation or implant or implantation or feces or faeces or microflora or fecal flora or faecal flora or transfusion or implant or implantation or instillation or donor or bacteriotherapy or infusion)) AND TS=(treatment or therapeutic or "reduction in recurrence" or successful or "reduction in rate")) AND TS=("ulcerative colitis" or UC)	633
